# Supplementary material for: Overcoming the Tropical Andes publication divide: Insights from local researchers on challenges and solutions
Source: PLoS One. 2024 Jun 26;19(6):e0306189. doi: 10.1371/journal.pone.0306189 (PMC11207134; doi:10.1371/journal.pone.0306189)
Supplement: S1 Appendix — Promotional materials and detailed itinerary for the 3-day scientific writing and publishing workshop. (PDF) [file pone.0306189.s001.pdf]

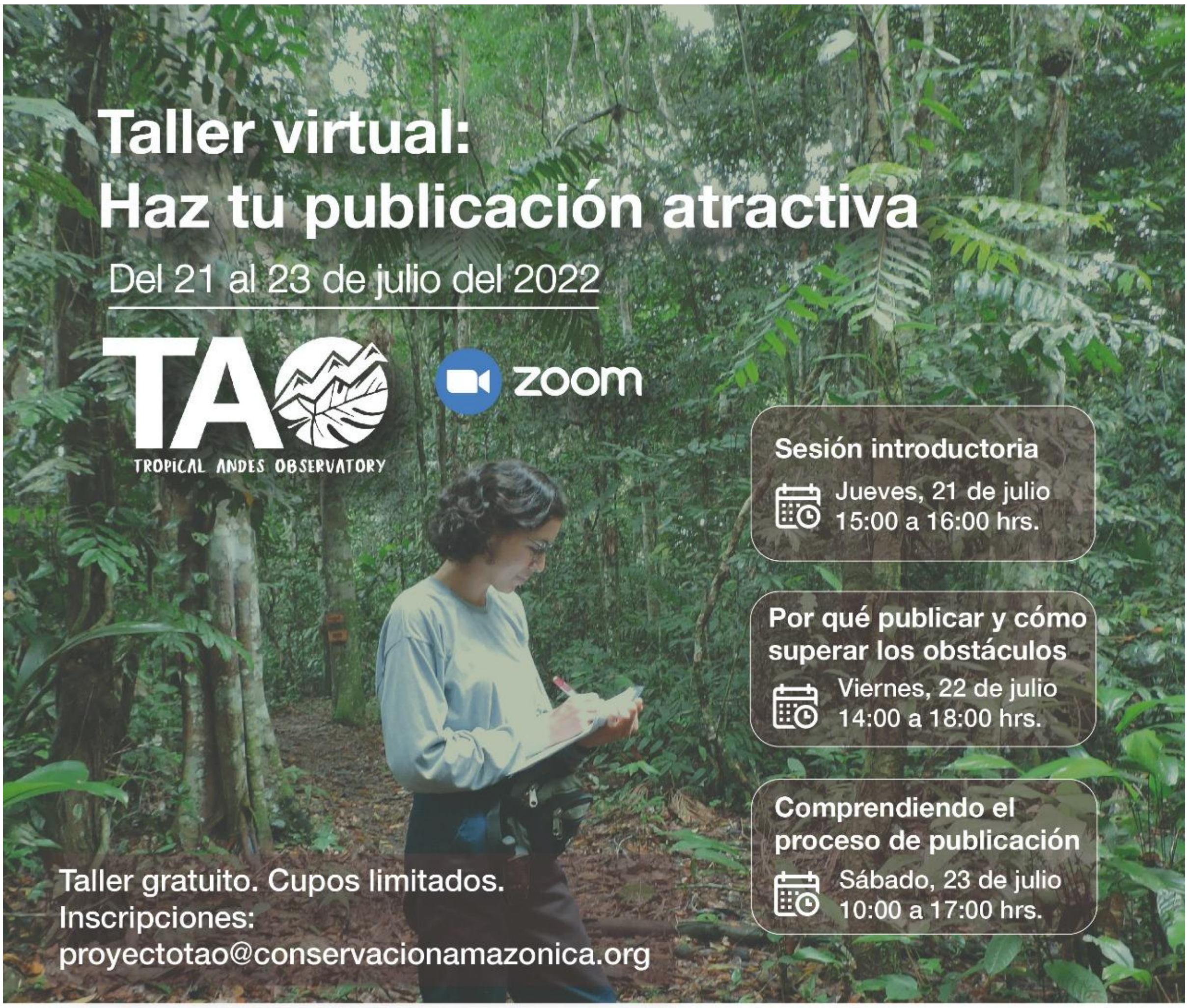

ACCA Conservación Amazónica   iDiv   Universidad de Córdoba

TALLER VIRTUAL: "Haz tu publicación atractiva"

PROYECTO TAO - DESDE LOS DATOS HASTA LAS DECISIONES

21 – 23 de julio, 2022

Lima - Perú

Presentación

Bajo la premisa de que las políticas públicas y procesos de toma de decisiones sobre desarrollo y manejo sostenible de los recursos naturales deben ser basados en ciencia e información de alta calidad, los socios del Proyecto TAO, liderados por el Centro Alemán para la Investigación de Biodiversidad Integrativa (iDiv) en Alemania y Conservación Amazónica – ACCA en el Perú, ponen a disposición de la comunidad de investigadores y gestores de biodiversidad de los países de los Andes Tropicales un taller diseñado para lograr publicaciones científicas efectivas.

El proyecto TAO ha identificado que, aunque una gran cantidad de investigaciones sobre biodiversidad y conservación que se publican están basadas en América Latina, un pequeño porcentaje es publicado por investigadores locales. Esta es una limitación importante que afecta la visibilidad, el impacto y la atención de los esfuerzos de conservación e investigación en la región.

Objetivo del Taller:

En este taller discutiremos por qué debemos publicar, cómo superar los obstáculos particulares de América Latina para lograrlo y abordaremos el proceso de publicación. Trabajaremos en transformar los datos e informes de los participantes en una historia convincente e interesante para su publicación en revistas científicas.

Programa

Jueves 21

15:00-16:00 Sesión introductoria

Viernes 22

¿Por qué publicar y cómo superar los obstáculos?

14:00-15:00 La importancia de publicar manuscritos científicos
15:00-16:30 Barreras comunes para los investigadores latinoamericanos y cómo superarlas
Pausa
16:45-18:00 Barreras comunes para los investigadores latinoamericanos y cómo superarlas

Sábado 23

Comprendiendo el proceso de publicación

10:00-11:00 Presentación previa: cartas de presentación, coautoría, formateo y selección de la revista adecuada
11:00-12:00 Postenvío: decisiones editoriales, comentarios de revisores y reenvíos
12:00-13:00 *Almuerzo*

Creación de un manuscrito científico

13:00-14:00 Convertir datos e informes en artículos científicos
14:00-15:00 Creación de una historia coherente e interesante
Pausa
15:15-17:00 Organizar un manuscrito científico utilizando datos e informes propios

Acceso Zoom para el Taller

Para aplicar por favor llene el formulario de inscripción haciendo click **aquí**, posteriormente recibirá un correo de confirmación con los enlaces Zoom correspondientes a cada sesión.

Para consultas, escríbanos al correo: proyectotao@conservacionamazonica.org
